# Lung Ultrasound to Monitor Disease Severity and Aid Prognostication in COVID-19 Pneumonia: A Retrospective Analysis of Serial Lung Ultrasound Assessments

**DOI:** 10.24908/pocus.v6i2.15195

**Published:** 2021-11-23

**Authors:** Matthew Llewelyn Gibbins, Quentin Otto, Paul Adrian Clarke, Stefan Gurney

**Affiliations:** 1 Anaesthetic and Intensive Care Medicine Consultant, Bristol Royal Infirmary (BRI) Bristol, BS2 8HW; 2 Acute Care Common Stem trainee, Bristol Royal Infirmary (BRI) Bristol, BS2 8HW

**Keywords:** Ultrasonography, Diagnostic imaging, Lung, Pneumonia

## Abstract

**Background: **The aim of this retrospective analysis was to assess if serial lung ultrasound assessments in patients with COVID-19 pneumonia, including a novel simplified scoring system, correlate with PaO_2_:FiO_2 _ratio, as a marker of disease severity, and patient outcomes. **Methods**: Patients treated for COVID-19 pneumonia in a tertiary intensive care unit who had a lung ultrasound assessment were included. Standardised assessments of anterior and lateral lung regions were prospectively recorded. A validated lung ultrasound score-of-aeration and a simplified scoring system based on the number of disease-free lung regions were correlated with: PaO_2_:FiO_2 _ratio, successful weaning from mechanical ventilation, and status (alive or dead) at discharge. MedCalc© statistical software was used for statistical analysis. **Results**: 28 patients (109 assessments) were included. Correlation was seen between score-of-aeration and PaO_2_:FiO_2_ ratio (r = -0.61, p<0.0001) and between the simplified scoring system and PaO_2_:FiO_2_ ratio (r = 0.52 p<0.0001). Achieving a score-of-aeration of ≤9/24 or ≥2 disease-free regions was associated with successful weaning from mechanical ventilation and survival to ICU discharge (accuracy of 94% and 97% respectively). **Conclusion**: Retrospective analysis from this small cohort of patients demonstrates that scores-of-aeration and a simplified scoring system based on the number of disease-free antero-lateral regions from serial LUS assessments correlate with PaO_2_:FiO_2_ ratio as a marker of disease severity in patients with COVID-19 pneumonia. In addition, lung ultrasound may help identify patients who will have favourable outcomes.

## Background

Point of care Lung Ultrasound (LUS) is validated in the diagnosis and monitoring of lung pathology^1^, ^2^, ^3^ and has proven a useful imaging modality during the SARS-CoV-2 (COVID-19) pandemic 4. The peripheral distribution of pathological changes within the lungs facilitate detection by LUS^5^ and early reports demonstrate good correlation with computerised tomography (CT) and a higher diagnostic yield than chest x-ray 4, 6, 7. Lung Ultrasound has additional advantages over other imaging modalities. These include immediate availability for point of care serial assessments, low cost, and its safety profile for both the operator and patient 4. 

The characteristic LUS features of COVID-19 pneumonia that have been described include; pleural line irregularities with subpleural consolidation, extensive ‘B-lines’ (vertical artefacts extending from the pleura) which affect most lung regions, and in severe cases dependant consolidation. Pleural effusions are uncommon and the reappearance of ‘A-lines’ (horizontal ‘reverberation’ artefacts) has been noted on resolution 6, 7, 8, 9.

During the COVID-19 pandemic reports have described the use of LUS in the diagnosis of COVID-19 pneumonia and for assessment of its severity and for assessing response to interventions such as prone positioning, recruitment manoeuvres and diuresis 4, 6, 7, 9. However, due to the need for rapid dissemination of experience and information, publications have been limited to narrative reviews or case studies with few patients and more importantly, limited comment on longitudinal data 10. 

The primary aim of this retrospective analysis was to establish whether findings from serial LUS assessments of patients admitted to critical care with COVID-19 pneumonia, including a novel simplified scoring system, correlate with Pa0_2_:Fi0_2_ ratio as marker of disease severity. Our secondary aim was to establish whether LUS findings correlate with patient outcomes.

## Methods

This study has been reported in accordance with the STROBE guidelines for observational studies (Figure S1) 11.

Pragmatic LUS assessment formed part of the standard care of patients with respiratory failure in our ICU during the COVID-19 pandemic.

A retrospective review of patients admitted to our tertiary ICU with respiratory failure during the global COVID-19 pandemic was performed. Patients with a confirmed diagnosis of COVID-19 pneumonia via polymerase chain reaction (PCR) from mucosal swabs (Cepheid Xpert Xpress SARS-CoV-2 assay) and a documented LUS assessment were included. Any patient with a high clinical suspicion and treated for COVID-19 despite negative PCR were also included. Patients were followed up until discharge from the ICU. 

Patients had a LUS assessment as soon as practical following admission to ICU, and at non-standardised intervals thereafter. The timings of assessments were determined pragmatically dependant on patient progress, availability of trained operators, patient condition, patient position and clinical questions relevant to their care.

Serial LUS assessments were performed by the first author (MG, accredited in LUS through the Intensive Care Society of the United Kingdom) or by one of seven additional operators and archived. All images, scores and findings from archived assessments were ratified by the first author retrospectively. Assessments were performed using a Venue Go™ Ultrasound System (GE Healthcare UK, Amersham, UK). A C1-5 curvilinear probe and software programs specific to LUS were used for all assessments; ‘Lung’ mode optimised image settings to distinguish B-lines, and ‘cons/effusion’ mode optimised image settings to identify collapse/consolidation and effusions. 

Standardised assessment of the lungs was made by dividing the thorax into 12 regions 12. All regions were assessed when possible. Each region was then assigned a score based on the worst findings within any ‘lung window’ during a single respiratory cycle, as follows: normal A-lines or a single B-line = 0, ≥2 separate B-lines = 1, coalescent B-lines = 2, and collapse/consolidation = 3. Pleural irregularity alone scored 0. The total LUS score of aeration was calculated by the sum of the individual scores and ranged from 0 to 36 13. The findings for each region, the total LUS score of aeration, patient position and PaO_2_:FiO_2 _ratio were prospectively entered onto a specific reporting proforma within the patient’s electronic clinical records (IntelliSpace Critical Care and anaesthesia, Philips, Guildford UK) when the operator completed each scan. Additional findings were documented as free text, where relevant. 

Observations from the initial analysis revealed a potential association between the number of ‘disease-free’ regions and clinical improvement/patient outcomes. A disease free regions was defined as: A-lines or a single well-defined B-line in all lung windows within that region (+/- isolated pleural irregularities), leading to an aeration score of zero. The relationship between the number of disease-free regions and PaO2:FiO2/patient outcomes was analysed to establish whether a binary outcome for each lung region (‘disease-free’ or ‘disease-present’) could be used as an alternative ‘simplified scoring system’ or SSS. 

The following baseline patient characteristics were retrieved from the Intensive Care National Audit and Research Centre (ICNARC) database; sex, age, ethnicity, very severe comorbidities, and disease severity scores at admission (APACHE II). The following patient outcomes were recorded; ICU length of stay, whether successful weaning from invasive ventilation was achieved and status (alive or dead) at ICU discharge.

Statistical analysis was performed using MedCalc© statistical software. Confidence intervals for the predictive values are standard logit confidence intervals and confidence intervals for accuracy are "exact" Clopper-Pearson confidence intervals. Correlation was assessed using Spearman’s rank correlation coefficient for non-normally distributed data, with 95% confidence intervals and P-values. DeLong et al methodology was used for receiver operating characteristic curve analysis.

## Results

**Figure 1:**
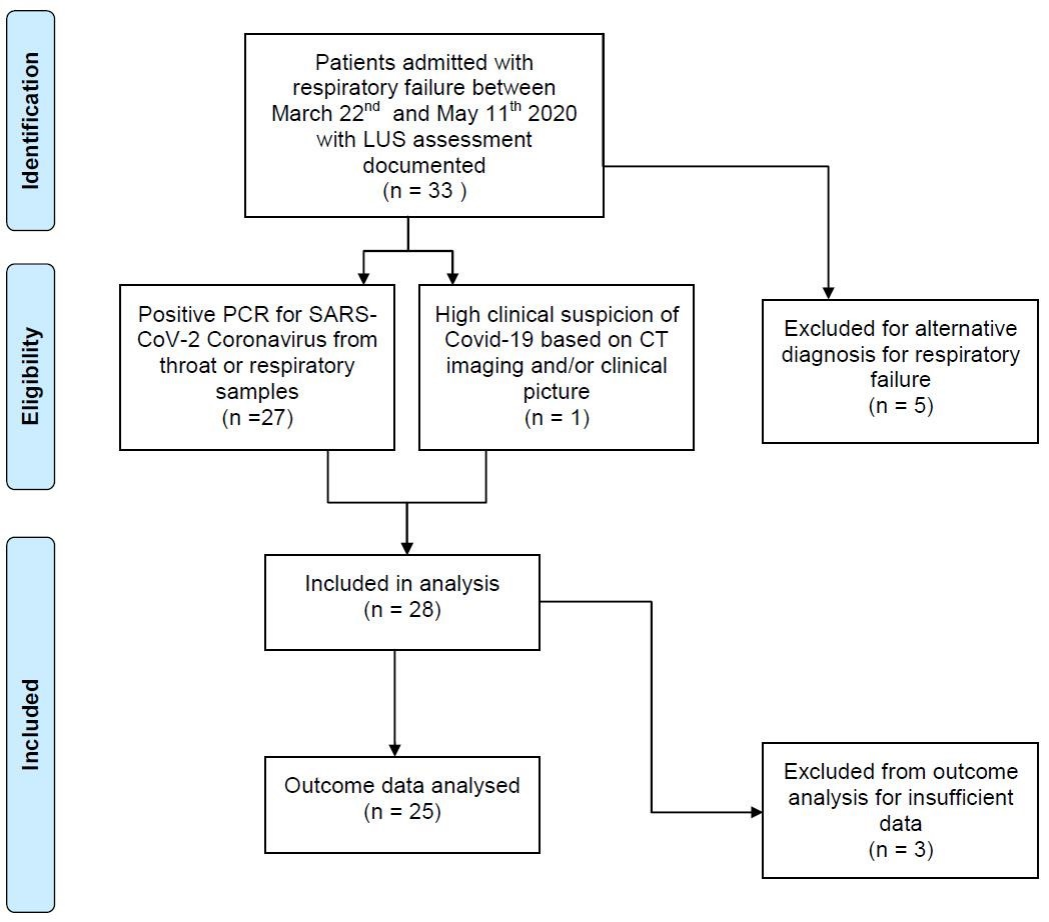
Flow diagram of patients included in analysis.

33 patients admitted to our ICU with respiratory failure between March 22^nd^ and May 11^th^, 2020 had a LUS assessment documented. 117 LUS assessments were performed - 79 (68%) were performed by the first author and 38 were performed by one of seven additional operators and the findings were ratified retrospectively. 28 patients had a clinical diagnosis of COVID-19 pneumonia and were included in initial analysis. 27/28 patients had a positive PCR from throat or tracheal swabs. The remaining patient was assigned the diagnosis of COVID-19 pneumonia based on chest CT and clinical findings. One patient was excluded from our analysis of correlation with PaO_2_:FiO_2 _ratio and a further two patients were excluded from analysis regarding outcomes, due to insufficient LUS data (Figure 1). 

Characteristics of the 28 patients are summarised in Table 1 and compared to patients included in the contemporaneous iteration of the United Kingdoms (UK) ICNARC report, published June 12^th^, 2020 14. 27/28 (96%) patients required invasive ventilation and the mean duration of admission was 18.5 days (range 2-52). 109 LUS assessments were performed, with a mean of four per patient (range: 1-10). 14 were performed in the prone position and 95 in the supine position. Classic features of COVID-19 pneumonia were seen in all patients, with diffuse B-lines and subpleural consolidation the most prominent features. The mean score (corrected) out of 36 for patients who were successfully weaned from invasive ventilation and survived to ICU discharge was 14.5, versus 20.6 for those who did not (difference of 6.1, 95% C.I. 9.8 to 2.5, p = 0.0021).

**Table 1:** Baseline patient characteristics and medical history/indicators of acute severity in the 28 patients included, compared to data obtained from the United Kingdom Intensive Care National Audit and Research Centre (ICNARC), published on 12th June 2020. APACHE II = Acute Physiological Assessment and Chronic Health Evaluation II.

Demographics	Study Cohort (N=28)	ICNARC Data (N=9777)
**Mean age (years) at admission (SD)**	61.7 (9.4)	58.7 (12.6)
**Sex, n (%)**		
Male	22 (78.6)	6908 (70.7)
Female	6 (21.4)	2864 (29.3)
**Race/Ethnicity, n (%)**		
White	13 (46.4)	6008 (67.1)
Mixed	3 (10.7)	154 (1.7)
Asian	2 (7)	1339 (15.0)
Black	4 (14.3)	869 (9.7)
Other	1 (3.6)	583 (6.5)
Not stated	5 (17.9)	n/a
**Body mass index, n (%)**		
<18.5	0	60 (0.7)
18.5-24.9	5 (17.6)	2292 (25.3)
25-29.9	10 (35.7)	3158 (34.8)
30-39.9	11 (39.3)	2856 (31.5)
40+	2 (7.1)	710 (7.8)
**Medical History/indicators of acute severity**	**Study Cohort (N=28)**	**ICNARC Data (N=9777)**
**Dependency prior to admission, n (%)**		
Able to live without assistance of daily activities	25 (89.3)	8589 (90.4)
Some assistance with daily activities	3 (10.7)	876 (9.2)
Total assistance with all daily activities	0	31 (0.3)
**Very serious co-morbidities, n (%)**		
Cardiovascular	0	59 (0.6)
Respiratory	0	106 (1.1)
Renal	0	160 (1.7)
Liver	0	40 (0.4)
Metastatic disease	0	46 (0.5)
Haematological malignancy	0	173 (1.8)
Immunocompromised	0	328 (3.4)
**Mean prior hospital length of stay in days (SD)**	1.0 (1.9)	2.5 (6.9)
**Mean APACHE II score (SD)**	14.6 (5.0)	14.9 (5.3)
**PaO_2_:FiO_2 _ratio (based on lowest PaO_2 _in first 24 hours), n (%)**		
≤13.3kPa (≤100mmHg)	10 (35.7)	3306 (37.2)
>13.3 and ≤26.7kPa (>100and ≤200mmHg)	10 (35.7)	4267 (48.0)
>26.7kPa (>200mmHg)	8 (28.6)	1309 (14.7)

Not all LUS assessments included all 12 regions. The majority of LUS assessments were performed with the patient in the supine position, all of which included eight anterior and lateral regions. To generate a standardised data set and allow meaningful correlation with clinical variables and outcomes, only findings from these eight antero-lateral assessments - giving a total worse possible score of aeration of 24 - were used for further analysis. This was not possible in 14 LUS assessments performed in the prone position. In total 95 LUS assessments from 27 patients (one patient was only assessed in the prone position and was therefore excluded) provided the scores of aeration and the number of disease-free regions for further analysis. 

### Antero-lateral scores/SSS and PaO_2_:FiO_2 _ratio 

**Figure 2:**
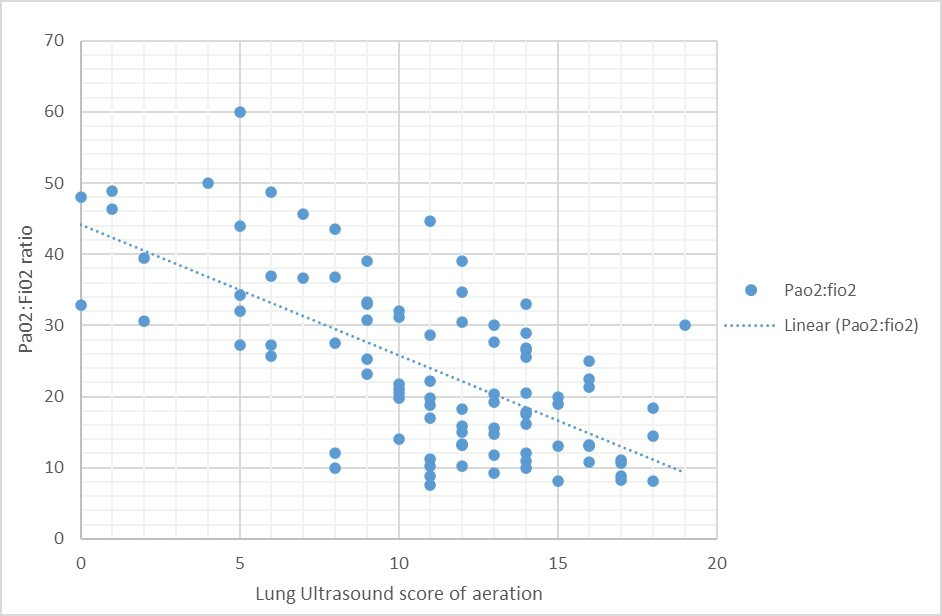
Graph demonstrating the correlation between lung ultrasound scores ofaeration and PaO2:FiO2 ratio

**Figure 3:**
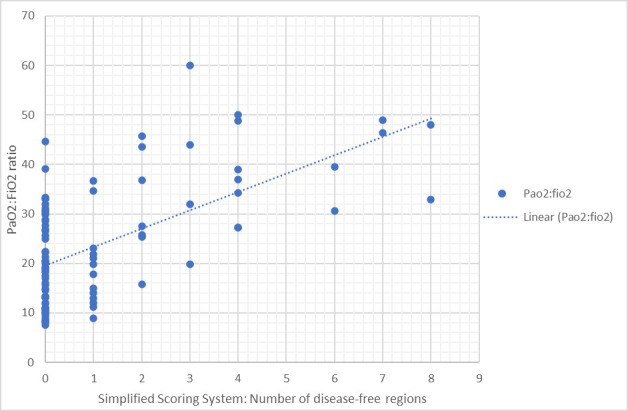
Graph demonstrating the correlation between the Simplified Scoring System (Number of disease-free regions) and PaO2:FiO2 ratio

From the 27 patients included, correlation was demonstrated between antero-lateral scores of aeration and PaO_2_:FiO_2 _ratio, r = -0.611 (95% C.I. -0.72 to -0.47 p<0.0001) Figure 2, and between the SSS number of disease-free regions and PaO_2_:FiO_2_, r = 0.52 (95% C.I. 0.35 to 0.65, p<0.0001) Figure 3. 

### Antero-lateral scores/SSS and patient outcomes

Table 2 summarises the relationship between lowest (best) antero-lateral scores ofaeration and the highest (best) number of disease-free regions achieved by each patient and patient outcomes for the 25 patients included (outcomes are awaited in one patient and one patient was excluded as only one assessment – on admission following inter-hospital transfer – was performed). Receiver Operating Characteristics (ROC) analysis demonstrated that an antero-lateral score of ≤9/24 or an SSS of ≥2 disease-free regions at any point during critical care admission were most accurate at identifying patients who successfully weaned from mechanical ventilation and survived to ICU discharge. An antero-Lateral score of ≤9/24 had a sensitivity of 100% and a specificity of 83%, giving a PPV of 92% (95% C.I. 76% to 98%), a NPV of 100% and an accuracy of 94% (95% C.I. 77% to 99%). The Area Under the ROC Curve was 0.93. For the SSS, ≥2 disease-free regions had a sensitivity of 100% and a specificity of 92%, giving a PPV of 95% (95% C.I. 77% to 99%), a NPV of 100% and an accuracy of 97% (95% C.I. 81% to 99.9%). The Area Under the ROC Curve was 0.97.

**Table 2:** Relationship between best (lowest) antero-lateral scores of aeration, highest number of disease-free regions and patient’s outcomes for the 25 patients included in the analysis.

	Lung ultrasound assessments		Outcomes	
**Patient No.**	**Best (lowest) Anterior-Lateral score of aeration**	**Highest number of disease-free regions**	**Successfully weaned from mechanical ventilation**	**Survived to ICU discharge**
1	8	2	Yes	Yes
2	6	3	Yes	Yes
3	6	4	Yes	Yes
4	12	0	No	No
5	9	2	Yes	Yes
6	16	0	No	No
7	5	3	No	No
8	2	6	Yes	Yes
9	10	0	Yes	No
10	12	0	No	No
11	10	1	No	No
12	14	1	No	No
15	16	1	No	No
16	0	8	Yes	Yes
17	13	0	No	No
18	11	1	No	No
22	8	2	Yes	Yes
23	6	5	Yes	Yes
24	5	4	Yes	Yes
25	8	2	Yes	Yes
27	17	0	No	No
29	5	4	Yes	Yes
31	9	4	n/a	Yes
32	6	4	Yes	Yes
33	9	0	Yes	No
				

14 patients were successfully weaned from mechanical ventilation. 13/14 (93%) patients met both the above criteria, seven of which (54%) did so prior to transitioning to a spontaneous ventilation mode. Four of the remaining six patients met both criteria prior to extubation. One patient met both criteria and did not receive ventilatory support at any point and one patient met the criteria after extubation. One patient achieved an antero-lateral score of 9/24 prior to extubation but did not demonstrate any disease-free regions and subsequently required re-intubation, not surviving to ICU discharge. 

## Discussion

This study demonstrates that findings from serial LUS assessments can be used to monitor severity of respiratory failure in patients with COVID-19 pneumonia, demonstrating correlation with PaO_2_:FiO_2 _ratio. The LUS score of aeration and SSS were also accurate at identifying patients with favourable outcomes.

The cohort of patients included is a predominantly representative sample. However, there is some divergence from the baseline characteristics of those included in the UK national database 14, particularly: patient ethnicity, mean prior hospital length of stay, and the proportion of patients with moderate and mild respiratory failure. The study included 95 individual LUS assessments, with over a third of patients having five or more during their ICU admission, providing valuable longitudinal data. Xing et al have published on the progression of LUS findings in COVID-19 pneumonia over a four-week period, however they included only 36 assessments and did not correlate findings with other clinical variables or outcomes 8.

The LUS score of aeration, developed by Soummer et al, is validated in the context of Acute Respiratory Distress Syndrome (ARDS) 13. Significant inter-individual variability, especially when differentiating separate from coalescent B-lines (a key component when generating the score of aeration) has been reported between experts in LUS 15, 16. This is likely to be exacerbated when performed by relative novices. The effect of interindividual variability in this cohort was minimised by ensuring all assessments included were performed or ratified by one clinician, however this approach is not pragmatic or scalable. COVID-19 pneumonia has been proposed to represent a distinct pathological process 17. The SSS has advantages in the context of a global pandemic. Owing to its binary nature it is likely to reduce interindividual variability and be easier to learn. 

Correlation between the SSS and PaO_2_:FiO_2 _was not as strong as that seen between the LUS score of aeration and PaO_2_:FiO_2_, however this requires confirmation. This may be because the simplified nature of the SSS does not record subtle fluctuations in loss of aeration which lead to worse PaO_2_:FiO_2_ ratios. The ability of the SSS to predict favourable outcomes appears comparable to that of the LUS score of aeration. A possible explanation for this is that patients that do not survive develop long term lung injury because of COVID-19 infection and therefore do not demonstrate return of ‘normal’ LUS findings or achieve ≥2 disease-free regions. Those that survive, experience resolution of lung injury to the extent that whole regions appear disease free on LUS, which the SSS is equally adept at identifying.

Posterior regions were excluded from the final analysis to provide a standardised data set. This represents a pragmatic approach to the use of LUS in the supine position. Moving ventilated patients, especially at a time when staffing levels were stretched, to assess all 12 regions would have compromised their safety. Although a standardised data set from the supine position was used, assessment of posterior regions remains important, for example when assessing for the potential benefit of prone ventilation 18, especially as COVID-19 pneumonia may demonstrate posterior predominance 19. 

As the LUS assessments were performed in a pragmatic fashion to aid in the management of critical ill patients, the timing of the LUS assessments was not standardised. Attempts were made to perform a LUS assessment at (or as close to) admission to critical care as possible, however this was not possible in all cases. A proportion (31%) of patients that achieved an antero-lateral score of aeration of ≤9/24 or an SSS of ≥2 disease free regions did so without ever achieving higher (worse) scores or fewer (worse) disease-free regions. In all these cases, the initial assessment was performed >7 days after admission to critical care. This was due to a lack of competent clinicians being available on/around the time of admission in two cases and in two cases patients were transferred from other hospitals (for capacity reasons) after prolonged critical care admission. It is not possible to ascertain if these patients had milder disease at admission to critical care or whether worse LUS scores were missed. However, whether due to more mild disease or resolution of severe disease, achieving an antero-lateral score of aeration of ≤9/24 or an SSS of ≥2 disease-free regions at any point during critical care admission was associated with a favourable outcome in our patient group.

This study has some significant limitations. This was a small, retrospective analysis from a single site. The disease being investigated is new, with rapidly changing management. The timing of our assessments were not standardised and our analysis excluded the posterior regions of the lungs. As with any point of care ultrasound assessment, there is potential for inter-individual variability in the interpretation of LUS findings. As a result, the findings of our analysis should be considered hypothesis generating.

## Conclusions

Our retrospective analysis of 95 serial LUS assessments in a cohort of 28 patients with COVID-19 pneumonia demonstrates that findings correlate with PaO_2_:FiO_2_ratio as a marker of disease severity and could provide useful information regarding prognostication. We believe this simple, safe, and inexpensive bedside investigation can contribute to the wider patient assessment and aid decision making during the global COVID-19 pandemic. The easily learnt simplified scoring system warrants validating in a larger cohort.

## Declarations

Ethics approval and consent to participate - Ethics approval waived by the institution as this was a retrospective analysis of anonymised data.

Consent for publication – Not applicable.

Authors’ contributions – MG contributed to all stages, implementing a LUS service, performing and verifying LUS assessments, collecting and analysing the data and drafting and editing the manuscript. QO contributed by performing LUS assessments, collecting and analysing the data and editing the manuscript. SG contributed to data analysis and drafting and editing of the manuscript. AC contributed to implementation of the LUS service and editing the manuscript. All authors read and approved the final draft of the manuscript.

## Competing interests and funding 

Non to declare

## Supplementary Material

Supplementary Figure S1 STROBE Statement—checklist of items that should be included in reports of observational studies.
